# Exploring atmospheric free-radical chemistry in China: the self-cleansing capacity and the formation of secondary air pollution

**DOI:** 10.1093/nsr/nwy073

**Published:** 2018-07-19

**Authors:** Keding Lu, Song Guo, Zhaofeng Tan, Haichao Wang, Dongjie Shang, Yuhan Liu, Xin Li, Zhijun Wu, Min Hu, Yuanhang Zhang

**Affiliations:** 1 State Key Joint Laboratory of Environmental Simulation and Pollution Control, College of Environmental Sciences and Engineering, Peking University, Beijing 100871, China; 2 CAS Center for Excellence in Regional Atmospheric Environment, Chinese Academy of Sciences, Xiamen 361021, China

**Keywords:** atmospheric chemistry, free radicals, new particle formations, OH, NO_3_

## Abstract

Since 1971, it has been known that the atmospheric free radicals play a pivotal role in maintaining the oxidizing power of the troposphere. The existence of the oxidizing power is an important feature of the troposphere to remove primary air pollutants emitted from human beings as well as those from the biosphere. Nevertheless, serious secondary air-pollution incidents can take place due to fast oxidation of the primary pollutants. Elucidating the atmospheric free-radical chemistry is a demanding task in the field of atmospheric chemistry worldwide, which includes two kinds of work: first, the setup of reliable radical detection systems; second, integrated field studies that enable closure studies on the sources and sinks of targeted radicals such as OH and NO_3_. In this review, we try to review the Chinese efforts to explore the atmospheric free-radical chemistry in such chemical complex environments and the possible link of this fast gas-phase oxidation with the fast formation of secondary air pollution in the city-cluster areas in China.

## INTRODUCTION

In the conurbation areas of China, high concentrations of primary pollutants (e.g. SO_2_, NO_x_, volatile organic compounds (VOCs), etc.) are emitted from both anthropogenic and biogenic sources and these primary pollutants are oxidized by ambient free radicals and then transferred into sulfates, nitrates, particulate organic matter and ozone; subsequently, high concentrations of secondary pollutants (e.g. ozone and fine particulate matter) are presented in the atmosphere, from which the produced fine particles could play a catalytic role in further heterogeneous oxidation reactions. Finally, the fast emission and fast oxidation would result in serious air pollution on the scale of city clusters. Since the serious air pollution in China is driven by atmospheric oxidation of primary pollutants from both coal-burning and petrol consumption, it is a more complicated chemical system than that of the air pollution that took place in the developed countries such as the ‘London smog’ and ‘Los Angeles smog’, which were largely linked to coal-burning and petrol consumption separately. The regional air pollution took place in China was thus named as the ‘Air Pollution Complex’ to underscore these chemically complicated air-pollution processes (e.g. complicated reactants, complicated oxidation pathways as well as complicated oxidation products) [1–3].

Since the study of ‘Los Angeles smog’ and the removal of CO on a global scale, it is generally known that all the primary gas pollutants were mainly removed through gas-phase oxidation via hydroxyl radicals (OH), nitrate radicals (NO_3_) and ozone (O_3_) [4]. The atmospheric oxidation capacity by OH is initiated by the photolysis of O_3_ and maintained by the reaction with VOCs to generate hydrogen peroxy radical (HO_2_) and organic peroxy radicals (RO_2_), which are then recycled into OH via nitric oxide (NO). The OH radical is terminated by reaction with NO_2_ to produce HNO_3_. The atmospheric oxidation capacity by NO_3_ is also initiated by O_3_ and terminated by reaction with VOCs as well as the heterogeneous uptake of its reservoir species: dinitrogen pentoxide (N_2_O_5_). In recent studies, it has been found that these highly oxidized nitrogen compounds can follow certain denitrification processes, which generate photoactive species such as HNO_2_ and ClNO_2_ and are further recycled to become OH radicals as well as NO_x_ [5,6].

**Figure 1.**
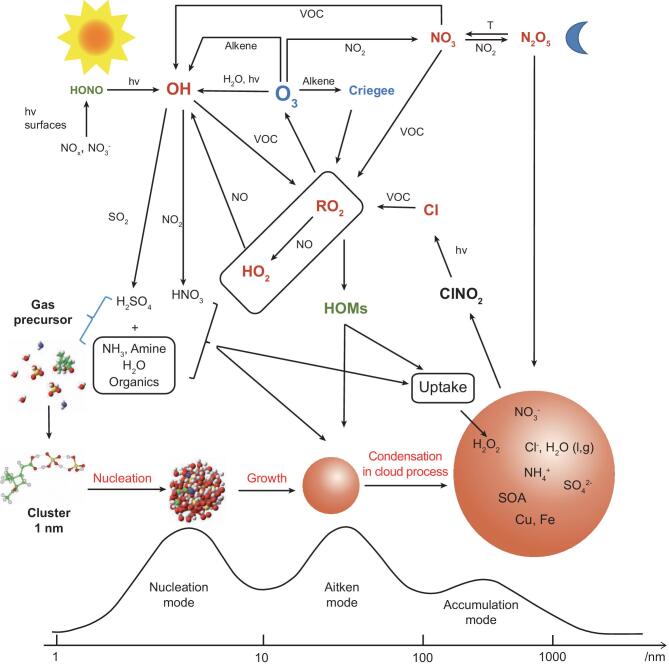
A schematic picture of the trace gas molecules’ complex transforming processes in the troposphere, which include the chemical reactions between the primarily emitted SO_2_, NO_x_, VOCs and the free radicals (OH, NO_3_) and the subsequent formation of secondary fine particles and O_3_.

In typical urban areas at China, the USA and Europe, driven by fast oxidation, the primary pollutants are transformed into low vapor pressure gas molecules such as sulfuric acid (H_2_SO_4_), nitric acid (HNO_3_) and highly oxidized organic molecules (HOMs). Assisted by ammonia (NH_3_) and water vapor (H_2_O) in the atmosphere, high concentrations of H_2_SO_4_ will enable fast gas-to-particle nucleation to take place, which then delivers a large amount of seed aerosols [7,8]. The high concentrations of HNO_3_ and HOMs will enable fast condensation onto seed aerosols and therefore a fast growth of fine particles will occur subsequently. These newly formed secondary particles have significant negative health and radiative impacts, which is a major factor that leads to an exceedance of the ambient air-quality standard in many countries. Moreover, the formed secondary particles can act as cloud condensation nuclei (CCNs) when the updraft condition appears. The water vapor condenses onto these particles during the updraft of the air masses and the CCNs become cloud droplets and will show an influence on climate change. In addition to the formation of secondary aerosols, a large amount of O_3_ is also produced in the fast oxidation of VOCs and NO_x_. O_3_ is known to have negative health impacts as well as a greenhouse gas. More importantly from a chemical perspective, O_3_ is the primary source of both the OH and NO_3_ radical as described above. So, overall, the whole atmospheric chemical system is autocatalytic with the presence of sunlight and primary pollutants (Fig. 1).

Due to the existence of such complex chemical reactions between pollutants and radicals (Fig. 1), many atmospheric processes are non-linear so that the mitigation of regional air pollution is not proportional to the mitigation of primary pollutant emissions. Since the ‘Los Angeles smog’, it has been well known that efficient pollution mitigation can only take place when a deeper understanding of the atmospheric chemical reactions is achieved prior to the action. With respect to the current situation in China, the ‘Air Pollution Complex’ is a kind of brand challenge in the field of atmospheric chemistry. The unprecedented chemical reactions among tens of thousands of different air molecules are complex, interesting and often beyond the current theory developed for London, Los Angeles and other major cities in both Europe and the USA. In this review, we will try to summarize the consensus efforts from both the Chinese and the international scientists toward the exploration of the unprecedented chemical reactions between the pollutants and the free radicals of the ‘Air Pollution Complex’ processes.

## MEASUREMENTS OF THE FREE RADICALS IN THE FIELD

The ambient radicals are extremely difficult to measure due to their high reactivity, short lifetime and low concentration. The high reactivity requires a low loss sampling method, the short lifetime requires a high time and spatial resolution, and the low concentration requires very high detection sensitivities and excludes tiny artificial radical production in the measurement instruments. None of these requirements is easy to fit experimentally. Through 20 years of efforts after the first establishment of the OH radical chemistry in the troposphere, the major technical breakthrough for the detection of OH, HO_2_ and NO_3_ radical was finally achieved in the early 1990s. Crosley (1995) [9] and Platt *et al.* (2002) [10] reported that several *in situ* measurement techniques such as LIF (Laser Induced Fluorescence), DOAS (Differential Optical Absorption Spectroscopy), CIMS (Chemical Ionization Mass Spectrometry) and MIESR (Matrix Isolation Electron Spin Resonance) had matured and become ready for field studies. In China, the research on the atmospheric radical chemistry was pioneered in the study of the photochemical smog in petrol industrial areas of Lanzhou, Gansu Province and Shanghai in the 1980s [11,12], led by Peking University. The initial efforts to detect OH in China were then conducted by the end of the 1990s of which three techniques such as LIF [13], EPR (Electron Paramagnetic Resonance) [14] and SC-HPLC (SCrubbing using salicyclic acid followed by high-performance liquid chromatography analysis) [15] were explored.

Continuous efforts on the measurement of the free radicals have been further conducted in China since the 2000s. A field-deployable LIF instrument for the detection of OH and HO_2_ was built in Peking University (PKU) as a joint effort of Forschungszentrum Juelich (FZJ) and PKU. A CIMS instrument for the OH detection was established in the lab in the Dalian Institute of Chemical Physics. The detection of peroxy radicals and the NO_3_ radicals was better established by several Chinese groups such as PKU, Anhui Institute of Optics and Fine Mechanics (AIOFM), Hong Kong Polytechnic University (HKPoly) with CIMS, DOAS and CEAS/CRDS techniques. The recent development progress of the HOx and ROx measurement techniques was nicely summarized by Stone *et al.* (2012) [32] and that of the NO_3_ radical by Brown and Stutz (2012) [33], respectively. According to these reviews and references therein, all the state-of-the-art measurement techniques used in recent field and chamber studies in China and worldwide have been summarized in Table 1. Since the establishment of the radical measurement techniques, extensive field measurements of atmospheric radicals have been abundantly conducted in the framework of comprehensive field campaigns since the middle of the 1990s [34].

**Table 1.** State-of-the-art measurement techniques for the detection of ambient HOx, ROx and NO_3_ radicals worldwide.

Techniques	Species	Principle	Chinese groups	International groups
LIF^d^	OH, HO_2_	OH: OH + hν(308 nm) → OH*→ hν HO_2_: HO_2_+NO→OH followed by LIF detection of OH	PKU	FZJ, MPI, Leeds, Lille, PSU, Indiana, JAMSTEC
	RO_2_	RO_2_(+CO/NO)→HO_2_^a^ followed by LIF detection of HO_2_	–	FZJ, Leeds
	NO_3_	NO_3_ + hν(623 nm) → NO_3_*→ hν	–	TMU [16]^e^
CIMS^d^	OH	OH+^34^SO_2_+M→H^34^SO_3_;	CAS (DICP) [17]	DWD, NUIG, Helsinki, CNRS, Colorado
		H^34^SO_3_+O_2_→^34^SO_3_+HO_2_,		
		^34^SO_3_+H_2_O→H_2_^34^SO_4_;		
		H_2_^34^SO_4_+NO_3_^−^ċHNO_3_→H^34^SO_3_^−^ċNO_3_+HNO_3_		
	HO_2_, RO_2_	RO_2_/HO_2_(+NO/SO_2_)→ H_2_SO_4_^b^ followed by CIMS detection of H_2_SO_4_	HKPoly	DWD, Helsinki, CNRS, Colorado, GIT [18]
DOAS^d^	OH	OH + hν(308 nm) → OH*	–	FZJ, Frankfurt Univ [19]
	NO_3_	NO_3_ + hν(623–662 nm) → NO_3_*	CAS (AIOFM) [20]	California [21]
CEAS	NO_3_	NO_3_ + hν(662 nm) → NO_3_*	PKU [22], CAS (AIOFM) [23]	NOAA [24], Cambridge [25], MPIC [26], Cork [27], UEA [28]
CRDs	ROx*	RO_2_/HO_2_(+CO/NO)→NO_2_^c^ followed by CRDs/CEAS detection of NO_2_	CAS (AIOFM) [29]	Bremen [30]

^a^RO_2_+NO→HO_2_; HO_2_+NO→OH; OH+CO→HO_2_.

^b^RO_2_+NO→HO_2_; HO_2_+NO→OH; OH+SO_2_→HO_2_+H_2_SO_4_.

^c^NO+RO_2_→HO_2_+NO_2_; NO+HO_2_→OH+NO_2_; OH+CO→HO_2._

^d^Revised version according to the report of the International HOx Workshop 2015 [31].

^e^Not applied in the field so far.

FZJ, Forschungszentrum Juülich; MPIC, Max-Planck Institute for Chemistry, Mainz; Leeds, University of Leeds; Lille, Universiteé de Lille; PSU, Pennsylvania State University; Indiana, Indiana University; JAMSTEC, Japan Agency for Marine-Earth Science and Technology; TMU, Tokyo Metropolitan University; DWD, German Meteorological Service; NUIG, National University of Ireland Galway; Helsinki, University of Helsinki; CNRS, The National Center for Scientific Research (Orleans); Colorado, University of Colorado; GIT, Georgia Institute of Technology; Frankfurt Univ, University of Frankfurt; California, University of California; NOAA, National Oceanic and Atmospheric Administration; Cambridge, University of Cambridge; Cork, University of Cork; UEA, University of East Anglia; Bremen, University of Bremen.

**Figure 2.**
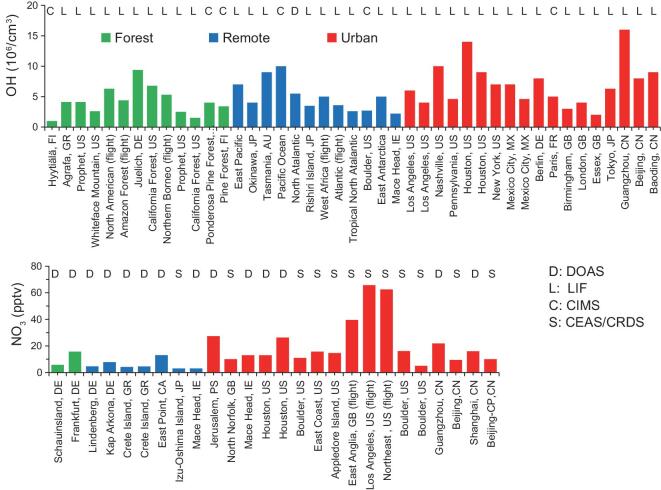
Typical observed daily averaged maximum OH concentrations and night-time averaged NO_3_ concentrations at distinct different geophysical regions (i.e. urban, remote, forest areas) with different measurement techniques (i.e. DOAS, LIF, CIMS, CEAS and CRDS). The summary of OH is an extension of Fig. 2 of Lu and Zhang [35], while that of NO_3_ is an extension of Fig. 3 of Wang *et al.* [36]. The results of Jiangmen (a rural site in Pearl River Delta) are unpublished observations.

According to the available observations, the OH concentrations always showed a pronounced diurnal profile following the change of the solar radiation and the variation between the peak value near noon and the night-time value could vary by more than two orders of magnitude. The peak value of the OH diurnal variation is considered to index the oxidation potential of a certain region. According to the modeling results, Finlayson-Pitts and Pitts (2000) [4] categorized the peak value of the OH diurnal profile into three typical geophysical conditions: continental land (10^5^–10^6^ cm^−3^), rural (10^6^–10^7^ cm^−3^) and urban (>10^7^ cm^−3^) areas. Herein, we adopted this way of classification for the available OH observations since 1990s. And we classified the observations into three categories: urban, remote (continental, marine, polar, free troposphere) and forested areas. The observed OH daily maximum concentrations in different categories are all in the range of 10^6^–10^7^ cm^−3^ (Fig. 2). The observations at urban areas, or more accurately strongly urban-influenced areas, showed a tendency of higher OH daily maximum concentrations, probably caused by the faster radical propagation from HO_2_ with the presence of the higher NO concentrations and the presence of higher O_3_ and OVOC concentrations. The highest OH concentration was once observed in PRD, with a value of 15 × 10^6^ cm^−3^. The HOx radical measurements in the urban areas were mostly done using LIF techniques, except that of Paris, which had been performed using CIMS [37]. For the remote and forested areas, the CIMS technique was used as frequently as that of LIF [38]. It is noteworthy that significant measurement interferences (5–50%) were newly discovered for the LIF techniques so that the previous detection of OH radicals has potentially been subjected to unaccounted-for positive biases [39,40].

Compared to that of OH, the variation of NO_3_ radicals was mostly driven by the variation of NO and suppressed by the sunlight so that significant concentrations only presented during the night and the temporal profile showed no periodical variation pattern. The averaged night-time concentrations of NO_3_ are utilized for a comparison among the areas of urban, remote and forested areas (Fig. 2). Higher NO_3_ was often presented in the residual layer above the urban areas during the flight campaigns where its production channel (NO_2_ + O_3_ → NO_3_) is promoted, while the destruction channel (NO_3_ + NO → 2 × NO_2_) is suppressed. In China, the NO_3_ observations were mainly conducted using the DOAS technique, while, in the USA and Europe, the CRDS/CEAS techniques were used. The later measurements are with much higher spatial resolution so that they can be better interpreted using a box model simulation constrained to simultaneous measurements of related parameters.

## MODELING STUDIES OF THE OH-HO_2_-RO_2_ RADICALS AND THE DIAGNOSIS OF LOCAL PHOTOCHEMICAL OZONE PRODUCTION

**Figure 3.**
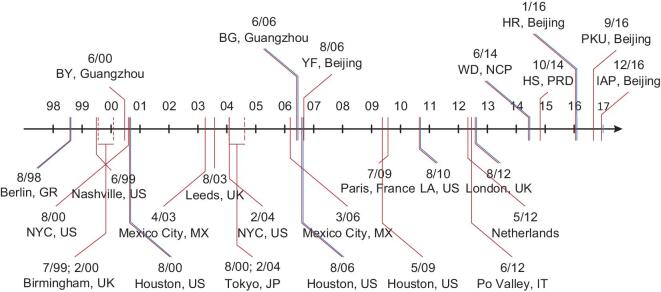
A timeline of HOx-ROx (marked as red labels) and NO_3_ (marked as blue labels) measurement campaigns performed in metropolitan areas since the end of the 1990s. The Chinese campaigns are placed at the upper part of the timeline, while the foreign campaigns are placed at the lower part.

Since the atmospheric radical reactions are very complex, as shown in Fig. 1, to explore the tropospheric radical chemistry often requires integrated field campaigns centered on radical measurements. As the HOx radical chemistry was first proposed at clean environments [41], comprehensive field campaigns were first made available for non-urban continental/coastal areas in the USA, Europe and Japan [34,42]. The preliminary results from those studies showed that the established chemical mechanism can describe the observed radical concentrations for the clean environments, though it was also demonstrated that the observations at mountain sites, which were influenced by biogenic emissions, showed some significant deviation between the observation and model results. Nevertheless, more challenging work started when we wanted to achieve quantitative understanding of the fast and complex chemistry running in the rural and suburban air influenced by nearby mega-cities. Closure studies on the major free radicals (OH, HO_2_, RO_2_ and NO_3_) were pioneered to some extent by the Berlin Ozone Experiment (BERIOZ) in summer 1998 at a rural site near city Berlin [43]. Afterwards, similar closure studies were performed in a number of large cities, such as New York [44], Birmingham [45], Nashville [46], Houston [47], Mexico City [48,49], Tokyo [50], Paris [37], Los Angels [51], London [52], the Netherlands (Zeppelin campaign) and Po Valley (Zeppelin campaign). In China, comparable integrated field campaigns were conducted at Beijing [53], Baoding (WD) [54] in the North China Plain and Guangzhou [55] (BG, HS) in the Pearl River Delta (see Fig. 3). Motivated by the serious winter haze and summer ozone pollution, seven comprehensive campaigns had already been performed in both rural and urban areas in China. According to the literature review, the recent efforts (specifically after 2012) in China gradually became the critical mass of research activities to investigate urban radical chemistry worldwide (Fig. 3). The other active radical measurement groups were currently focused on the scrutiny of the radical chemistry in forested areas.

**Figure 4.**
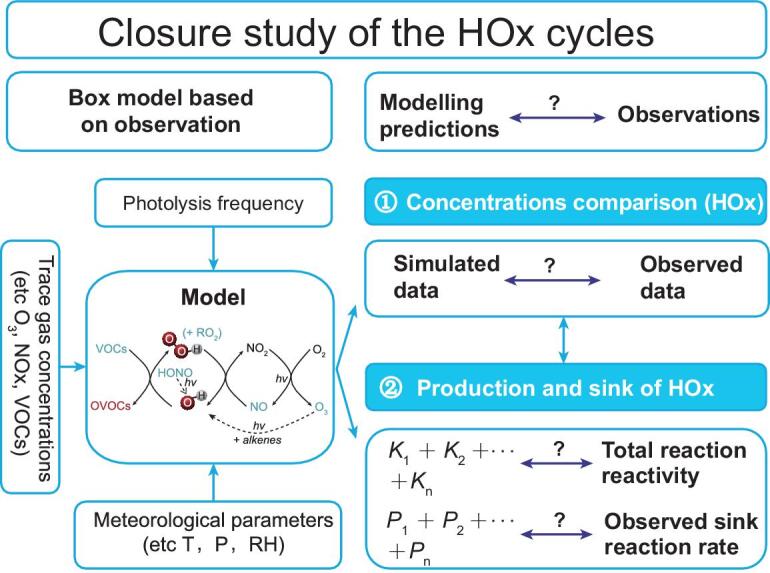
Sketch of the closure experiments for the exploration of HOx radical chemistry. The closure experiments include two types of model-observation comparisons: one is the comparison of concentrations and the other is the comparison of the reaction rates (production and sink).

The design of the integrated field campaigns can be guided by a simple principle—to enable the closure study of the radical cycles (e.g. HOx cycle; see Fig. 4). The concept of a closure study that requires an over-determined set of observations was first proposed in the aerosol characterization experiments. In the framework of closure studies for the HOx cycles, the target parameters—the ambient OH and HO_2_ concentrations, and the total OH reactivity—are measured directly and also calculated from a box model constrained by comprehensive observed parameters such as photolysis frequency, temperature, pressure, humidity and trace gas compounds. The comparison of the modeled and observed results of the target parameters thus provided a direct coherent evaluation of the current models. When a consistency of the measurement and model results is achieved within the accepted level of combined uncertainties of the model and measurement results, the model that represents the state-of-the-art knowledge on the tropospheric chemical mechanism is considered to be capable of delivering a reasonable description of the chemical reaction systems of the characterized air samples. Therefore, the validated tropospheric chemical mechanism can be further safely used in the higher-order models (e.g. air-quality model) for the diagnosis or forecast of activities of the regional air pollutions.

**Figure 5.**
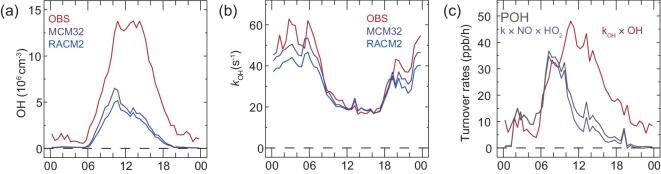
Application of the closure experiment for the exploration of OH chemistry during the Pearl River Delta 2006 campaign. (a) Comparison of the observed OH concentrations and that calculated from the observational constrained box model with MCM3.2 and RACM2 mechanisms, respectively, a modified version of [55]; (b) comparison of the total OH reactivity, a modified version of [58]; (c) comparison of the OH production rates (POH) and destruction rates (k_OH_ x OH), a modified version of [56].

The closure study of the HOx cycle was first realized in China during the Pearl River Delta 2006 (PRD2006) campaign [56,57]. In the first type of closure experiment shown as Fig. 5a, the observed OH concentrations are compared with the calculated concentrations with a state-of-the-art model constrained by independent measurements. This closure experiment is to test the capability of current chemical mechanisms (e.g. RACM2, MCM3.2) to model ambient OH concentrations. In the case of PRD2006, strong underestimation of OH by a factor of 3–5 for the afternoon hours is found for the current chemical mechanisms. Since OH is an extremely short-lived species, its concentrations reflect the ratio of its production and destruction. The strong underestimation of the concentrations can be either an overestimation of its destruction rate or underestimation of its production rate. To resolve this problem, the direct observation of the pseudo first-order reaction constant toward OH (k_OH_) was made available during field campaigns of about 15 years ago. The experimental determination of k_OH_ is a significant advancement in gas-phase chemistry that enables the second type of closure experiment shown as Fig. 5b. The *in situ* measured k_OH_ as determined by the first-order OH decay rate in the flow tube represents the total reactivity of the atmosphere toward that of OH. The calculated k_OH_ includes contributions from observed NO_x_, CO, VOCs, OVOCs and modeled OVOCs. The comparison of modeled and observed k_OH_ is useful to answer a trivial but important scientific question of whether we have measured all the important VOCs and OVOCs. Of PRD2006, the second type of closure experiment validates that the OH destruction part in the model is acceptable during the daytime [58]. At this point, we already know that the model strongly underestimates the observed OH concentrations and that is because of the strong underestimation of the OH production in the encountered air masses of PRD. Moreover, we can quantify the amount of the missing OH production rate through the comparison of the OH production and destruction rates, since these two terms should agree due to the extremely short lifetime of OH (<0.05 s in PRD). According to current knowledge, the total OH production rate (POH) consists of the sum of the OH primary production rate (photolysis of O_3_ and HONO, ozonolysis of alkenes) and secondary production rate (dominated by HO_2_ + NO). Calculation of the total OH destruction rate is much more complicated than that of the production rate, which includes tens of thousands of terms, mainly due to the complex of the ambient VOCs. Nevertheless, the calculation becomes quite simple after the direct determination of k_OH_ so that the term is equal to the product of OH and k_OH_. As depicted by Fig. 5c, the unknown OH production rate is about 30 ppb/h around noontime in PRD, which is three times larger than the known OH production rate.

**Figure 6.**
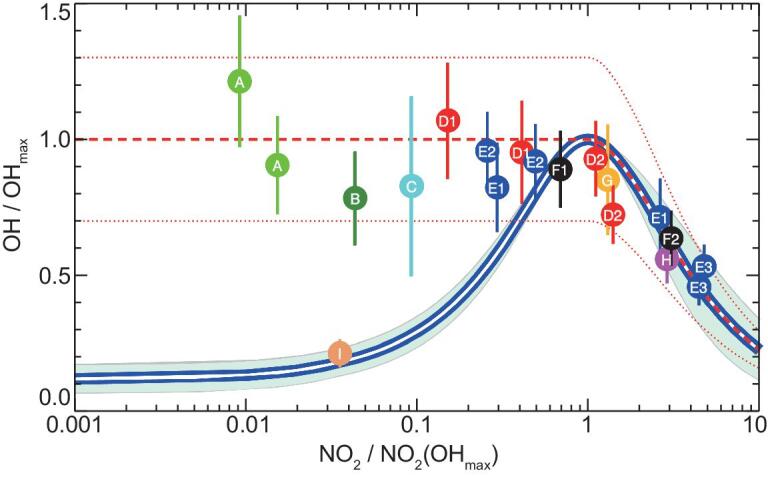
Comparison of observed and calculated OH concentrations for high VOC environments. NO_2_ and OH concentrations are normalized as explained in the text. Letters denote results from thirteen campaigns with hydrocarbon reactivity higher than 10 s^−1^: (A) Amazonia averaged daytime and afternoon hours (2005); (B) Borneo (2008); (C) US deciduous forest (1998); (D1) Pearl River Delta (2006) morning and afternoon hours; (D2) Heshan (Pearl River Delta, 2014) morning and afternoon hours; (E1) Beijing (North China Plain, 2006) morning and afternoon hours; (F1) Mexico City (2003); (G) Tokyo (2004); (H) New York (2001); (F2) Mexico City (2006); (I) US ponderosa pine forest (2009); (E2) Wangdu (North China Plain, 2014) morning and afternoon hours; (E3) Huairou (North China Plain, 2016) morning and afternoon hours. Error bars denote stated accuracies. The light-blue shaded area denotes the range of model results for individual campaigns and the full white/blue line denotes the average of all model calculations. The thick dashed red line represents the new concept and the dotted red lines denote an error estimate of ±30%, which is a typical value for the observational constrained box model calculations of OH.

The study in PRD and another study performed for Amazonia forest open up a general question of the current tropospheric chemical mechanism—where does the OH come from at high VOC environments (e.g. k_OH_ > 10 s^−1^)? Two kinds of answers were presented to this general question according to the published results. The campaigns performed at urban environments demonstrated that the OH comes from the reaction of HO_2_ plus NO, the photolysis of O_3_ and HONO as well as the ozonolysis of alkenes. The campaigns performed at forested areas showed that the OH comes from the photolysis of O_3_, the reaction of HO_2_ plus NO, and a so-far unknown OH-regeneration mechanism from peroxy radicals (e.g. isoprene-RO_2_ + HO_2_ → 2OH). The PRD2006 campaign is a kind of hybrid situation between urban and forested areas. The OH concentrations in the morning hours were sustained by the known urban type of sources, while the OH concentrations in the afternoon hours were amplified mainly due to the unrecognized OH-regeneration mechanism (e.g. RO_2_ + X → HO_2_ + X → OH). In a retrospective analysis of all the campaigns with high VOC reactivity (see Fig. 6), the published observations of OH at both urban and forest regions were reanalysed in one theoretical framework (dependence of OH on NO_2_). In this theoretical framework, all the OH observations performed in forested areas and observation at PRD2006 are found to be much larger than the corresponding model results as previously published and the observed OH concentrations are predictable by the maximum attainable OH concentrations through the change of imposed NO_2_ concentrations of the model. Moreover, closure study on the OH concentrations in recent field campaigns at North China Plain (i.e. Wangdu [54] and Huairou, unpublished results) and PRD (i.e. Heshan, unpublished results) is also realized in this framework. In these new campaigns, most of the observed OH concentrations can be explained by current models, while the modeled OH under low NO_x_ conditions showed underestimation of the observed values, which was however in the limit of the combined uncertainty levels of the observation and model calculations. New chemistry that regenerates more OH during isoprene degradation was indeed found through *ab initio* calculations and validated in chamber experiments. However, the validated new isoprene chemistry only showed a minor impact on the urban OH (<30%) due to the small contribution of the isoprene toward the total OH reactivity in the investigated atmosphere in China. Another explanation for the higher-than-expected OH concentrations is the OH measurement interference uncovered for certain types of LIF instruments. For example, the observed OH concentrations after correction of the measurement interference were nicely reproduced by the current models for two recent forest campaigns performed in the USA [39]. The discussion of the new chemistry for the high VOCs and low NO_x_ environments will continue but the first priority as a joint consensus of the HOx measurement community is to perform more systematic lab characterization on the possible OH measurement interference and to have intercomparison among different types of instruments in the near future.

**Figure 7.**
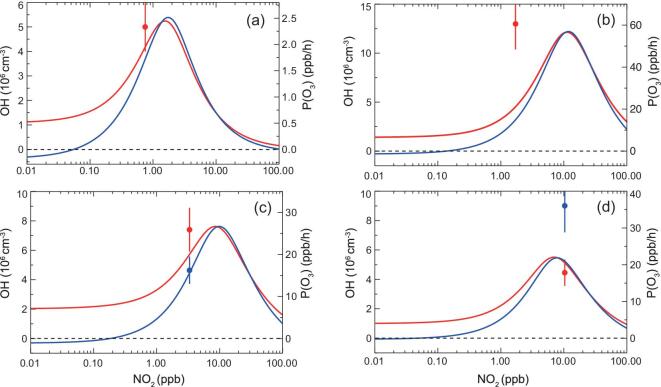
Dependence of the OH concentration (red line) and the net ozone production rate (P(O_3_) (blue line) on NO_2_ calculated with a steady-state model with typical conditions of rural Germany (a), Pearl River Delta (b) and North China Plain (c) and (d). Red and blue circles denote the observed values of OH and P(O_3_), respectively.

The above closure studies on the OH radical concentrations depict that the uncertainty of predicting OH for the high VOC and low NO_x_ conditions is a major problem of the current tropospheric chemical mechanisms. The implication on the formation rate of the secondary pollutants (e.g. local ozone production rate) is worth exploring also. In a classical picture of the photo-oxidation of the trace gas compound in the troposphere (Fig. 7a), a strong non-linear dependence of OH on NO_2_ is diagnosed. In the low NO_x_ range, the OH concentration will increase as the NO_2_ due to the reaction of HO_2_ + NO increases. In the high NO_x_ range, the OH will decrease as the NO_2_ due to the reaction of OH + NO_2_ increases. The dependence of the net local ozone production rate (P(O_3_)) on NO_2_ shows a close link with that of OH. For the remote areas, the observed OH concentrations can be nicely reproduced by the model. And the maximum P(O_3_) is only about 2 ppb/h due to the lack of VOCs. For the strongly urban-influenced conditions in China (PRD2006), the model largely underestimated the observed OH concentrations as discussed above (Fig. 7b). Nevertheless, the modeled local P(O_3_) is probably acceptable according to comparison of the base model and the model including the diagnosed new OH-regeneration mechanism (RO_2_ + X → HO_2_ + X → OH). Nevertheless, the experimental evidence is only limited to the good agreement of the observed and modeled HO_2_ concentrations. The impact on the modeled RO_2_ concentrations of the underestimated OH problem can not be fully explored due to the lack of RO_2_ measurement in PRD2006. In a recent campaign performed at a rural site (Wangdu) in the North China Plain, a full set of measurements of OH, HO_2_ and RO_2_ radicals were available for the first time in the field measurements in China. The new OH measurements were underestimated by the current model again for the low NO_x_ conditions (Fig. 7c). Nevertheless, the difference of the observed and modeled OH concentrations was in the levels of the combined uncertainties. The experimentally determined P(O_3_) was nicely reproduced by the current model mechanism for this condition as well. The new OH measurements were reproduced by the current model again for the high NO_x_ conditions, as in other urban studies (Fig. 7d). Nevertheless, the model strongly underestimated the experimentally determined P(O_3_) almost by a factor of two when the NO_x_ was high. The findings herein based on the observed HO_2_ and RO_2_ concentrations provide direct evidence of the long-discussed problem of the P(O_3_) underestimation solely based on the observed HO_2_ concentrations for the urban plumes. One possible explanation toward the underestimation of P(O_3_) at high NO_x_ air masses is the photolysis of ClNO_2_. But that channel only accounts for less than 10% as diagnosed for the conditions of Wangdu.

## GAS-PHASE OXIDATION AND NEW PARTICLE FORMATION

**Figure 8.**
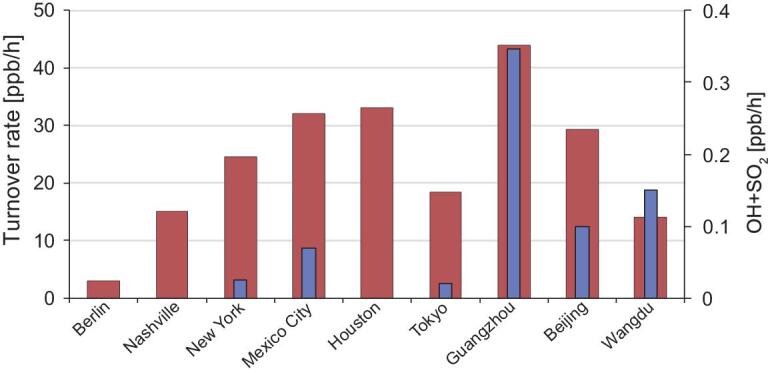
Turnover rate of the trace gas compounds (= OHxkOH, the red bars) determined in previous campaigns at different large cities worldwide. The calculated reaction rates of OH+SO_2_ (the violet bars) from observed concentrations of OH and SO_2_ are also compared for a few cities when reported.

Gas-phase oxidation of SO_2_ and VOCs will produce low vapor pressure molecules, such as H_2_SO_4_ and HOMs. These molecules can nucleate to form ∼1-nm particles. Thus, fast gas-phase oxidation would result in new particle formation (NPF) events, and influence air quality. The first step of particle formation processes is therefore determined by the gas-phase oxidation discussed above. The experimentally determined gas-phase oxidation rates as shown by OH times k_OH_ among different locations worldwide are compared in Fig. 8. The air masses characterized by campaigns in China are clearly subjected to fast gas-phase oxidation, which is similar to these values determined in cities at North America and Japan but much larger compared to the remote atmosphere (∼1 ppb/h) as well as that of European cities (represented by the case of Berlin observations). Nevertheless, much higher SO_2_ concentrations are presented in the current Chinese atmosphere due to the coal-based power generation. Consequently, a much faster reaction rate of SO_2_ by OH is presented in China than those in North America and Japan. Thus, new features of NPF are expected for the Chinese air masses with the co-presence of the fast turnover rate of trace gas compounds and the fast production of low-pressure compounds.

Features of ambient NPF events were investigated through long-term and intensive measurements. Long-term measurements of Particle Number Size Distributions (PNSD) with an SMPS (scanning mobility particle spectrometer) have been available since the 1990s, such as Hyytiälä, Finland [59] and Mace Head, Ireland [60]. The measurements of NPF in China started from 2004 when Wehner *et al.* (2004) first reported observing NPF events in Beijing [61]. Since then, the Peking University Urban Atmosphere Environment MonitoRing Station (PKUERS) has conducted the longest measurements of NPF in urban Beijing [62]. Continuous measurements of NPF were also made at the regional background site of Shangdianzi (SDZ), characterizing the NPF in rural NCP since 2011 [63]. The Station for Observing Regional Processes of the Earth System, Nanjing University (SORPES-NJU), has performed long-term observation of NPF in background air of YRD since 2011 [64]. In PRD, PNSD have been measured continuously since 2012 at the Guangdong Atmospheric Supersite [65]. NPF events in free troposphere at the Mt. Tai site have also been also observed permanently [66]. Those long-term measurements provided a brief understanding of NPF events in China, such as the seasonal variation and their relationship with other meteorological parameters like temperature and RH.

**Figure 9.**
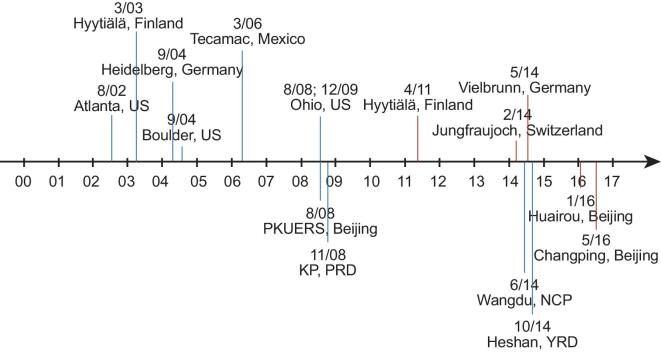
A timeline of campaigns aiming at NPF with sulfuric acid and HOMs measurements in addition to PNSD measurements around the world. Campaigns with sulfuric acids measurements are marked with blue lines, campaigns with both sulfuric acid and HOMs measurements are marked in red lines. The Chinese campaigns are placed at the lower part of the timeline, while the foreign campaigns are placed at the upper part. [67,73–77].

In addition to long-term measurements, intensive measurement campaigns with length of 1–2 months have also been performed globally. Figure 9 lists the intensive monitoring campaigns all over the world. In those intensive campaigns, advanced instruments were deployed, to get critical parameters in analysing mechanisms of nucleation and growth of particles. To achieve direct measurement of nucleation that started from 1 nm [67], a Particle Size Magnifier (PSM), Neutral cluster and Air Ions Spectrometer (NAIS) were used to measure the PNSD of 1- to 3-nm particles and 0.5- to 40-nm ions. Xiao *et al.* (2015) [68] and Yu *et al.* (2016) [69] reported NPF with sub-3-nm PNSD data in YRD, and analysed the formation rate (FR) and size-dependent growth rate (GR) of nanoparticles. CIMS was employed to detect the atmospheric content of sulfuric acid [70], organic acids and amine [71] in intensive campaigns globally (Fig. 9). Zheng *et al.* (2011) reported that the content of ambient H_2_SO_4_ during NPF in Beijing was 5 × 10^6^ cm^−3^, comparable with other foreign studies [70]. The concentration of ammonia and total concentration of certain amines—CH3NH2, C2H7N and C3H9N—were measured to be 1.7 ± 2.3 ppb and 7.2 ± 7.4 ppt in YRD [71]. In China, the CAREBeijing campaign (2008) [72], Wangdu campaign (2014), Huairou campaign (2016), Changping campaign (2016) in NCP and Heshan campaign in PRD integrated the measurement of critical precursors (OH, H_2_SO_4_ or HOMs) together with traditional PNSD measurement.

**Figure 10.**
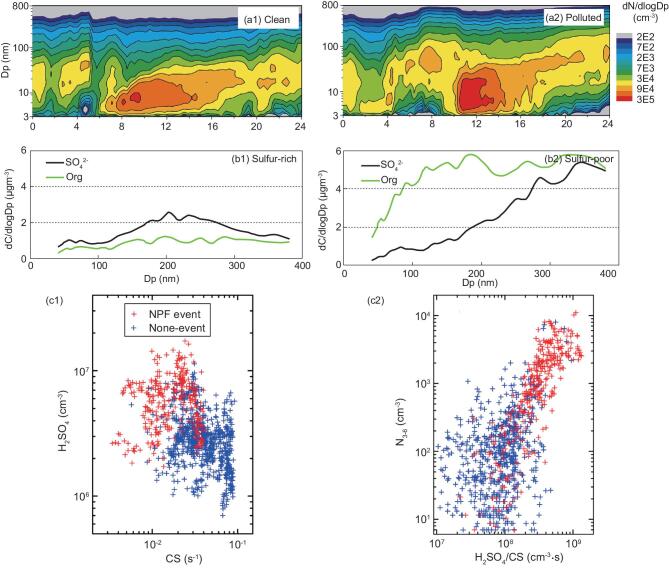
Typical particle number size distribution of polluted (a1) and clean (a2) types of new particle formation events observed in Beijing [62]; size-resolved mass distribution of sulfate (black line) and organics (green line) in particles during sulfur-rich (b1) and sulfur-poor (b2) particle growth events [78]; the relationships between (c1) sulfuric acid concentrations and condensation sink and (c2) the number concentration of 3- to 6-nm particles and the ratio of sulfuric acid concentration to condensation sink. The data are 10 min integrated between 08:00 and 11:00 of monitoring days during CAREBeijing2008 [72]. Data of NPF event days and Non-event days are distinguished as red and blue crosses, respectively. The NPF event was defined as: a new particle mode formed from 3∼6 nm, with higher number concentration compared with background particles; this new particle mode existed for more than 2.5 hours; the particle mode had a clear trend of growth. Days without NPF events were defined as ‘Non-event days’.

Based on the studies conducted in recent years, unique characteristics of NPF under complex air pollution and high oxidation capacity in China were revealed. First, besides the clean type (‘banana’ type, Fig. 10a1) NPF, which is widely observed in the clean areas, a distinct polluted type (‘apple’ type, Fig. 10a2) of NPF was observed in Beijing with a higher level of condensation sink (CS) (∼0.038 s^−1^) [62]. As a result of the fast oxidation of SO_2_ and VOCs, the concentration of particles burst on the whole range of 3–20 nm, with no trend in growth. The growth process can also be classified into ‘sulfur-rich’ (Fig. 10b1) and ‘sulfur-poor’ types (Fig. 10b2), in which sulfate and organics dominate in the particle composition, respectively.

Second, according to previous studies, the NPF events in China can only be observed when CS is lower than ∼0.05 s^−1^ (Fig. 9c1), while H_2_SO_4_ shows no obvious high value. The results indicated that the precursor and oxidation capacity in atmosphere of China are abundant, and CS is the constraint factor [31]. In polluted conditions where precursors are abundant, such as in winter of Shanghai, NPF can also occur with CS at around 0.1 s^−1^ [68].

Third, the high nucleation rate in polluted atmosphere can hardly be explained by classical homogenous nucleation theory in which sulfuric acid is the only precursor. Especially in Beijing, nucleation can be more efficient than other clean atmosphere studies under same level of H_2_SO_4_. The cluster activation and kinetic nucleation mechanisms need the exponent between FR and H_2_SO_4_ content to be around 1 or 2 [79,80]. However, in almost half of the NPF in Beijing, the exponent was higher than 2.5, as shown in Table 2 [72]. This indicated that, under high levels of anthropogenic VOCs and high oxidation capacity, thermodynamic nucleation including HOMs as precursor is important in China. Also, studies indicated that the dust-induced heterogeneous photochemical processes would enhance the formation of oxidants, and further promote the NPF process [81,82].

**Table 2.** Correlation exponent between gaseous H_2_SO_4_ and formation rate (FR) of new particle formation events in different environments.

	Hyytiälä	Heidelberg	Hyytiälä		Beijing
	QUEST II	QUEST III	BACCI/QUEST IV	Kent, Ohio	CAREBeijing 2008
*n* ∼ 1	38%	60%	45%	36%	12%
*n* ∼ 1.5	25%	30%	10%	36%	12%
*n* ∼ 2	31%	10%	30%	18%	18%
*n* > 2.5	6%	–	15%	9%	58%
Ref.	Riipinen *et al.* (2007)			Erupe *et al.* (2010)	Wang *et al.* (2011)

Lastly, due to the fast oxidation of gaseous pollutants, NPF events in China have stronger impacts on air quality and climate compared to clean atmosphere globally. The efficient nucleation in polluted atmosphere greatly contributes to the number concentration of CCN, as well as haze formation. Comprehensive field measurement showed that the haze formation typically includes two distinct secondary aerosol formation process, namely efficient nucleation, and fast and continuous growth. As shown in Fig. 9, the FR are relatively higher in Beijing [83] compared to the atmosphere in other environments, indicating more efficient nucleation. GR in Beijing have relatively larger variation, indicating more complicated growth mechanisms. The efficient nucleation process forms 10^3^∼10^4^ cm^−3^ nanoparticles that provide ‘seeds’ in the atmosphere. These ‘seeds’ rapid and continuously grow under high oxidation capacity in polluted atmosphere, and remarkably contribute to CCN and particle mass. Some studies showed that the growth of newly formed particles at the North China Plain could increase CCN concentration by 300% in 1 day [84]. The fast growth could also increase the particle mass by more than 200 μg·m^−3^ day^−1^, resulting in severe haze in the next 2–4 days [85].

Despite the current understanding, mechanisms of NPF in China triggered by the fast oxidation of gaseous pollutants are still ambiguous. Further work in investigating NPFs in China should include: (i) Obtaining more NPF parameters in various environments. Long-term measurements of PNSD should be maintained and conducted in various environments. Since the measurement of sub-3-nm particles is mature, it should be included in long-term measurements. More comprehensive monitoring studies should be conducted, integrating the measurement of PNSD and low-pressure gaseous precursors (H_2_SO_4_, HOMs, etc.); (ii) Developing and applying the technique in analysing NPF. Measurement of the chemical composition of the nucleation mode particles is the key to understanding the growth mechanisms, but it is still under development. Currently, particle hygroscopicity and density detected by the Hygroscopicity Tandem Differential Mobility Analyzer (H-TDMA) [86] and Aerosol Particle Mass (APM) analyser were used to estimate the possible composition of nanoparticles. (iii) Applying model simulation in NPF studies. Empirical models were established to simulate nucleation and growth or particles. Wang *et al.* (2013) and Huang *et al.* (2016) used the observational constrained box model to simulate the NPF events at PKUERS and SORPES-NJU station. The closure studies on the NPF showed that, except for H_2_SO_4_, oxidized organics could also take part in particle growth [87,88]. Based on the results from advanced instruments, the models should be improved and applied in China air masses.

## CONCLUSIONS AND OUTLOOK

To explore the atmospheric free-radical chemistry in the troposphere playing a central role in the study of tropospheric chemistry, regional air pollution and global climate change, the measurement of the atmospheric free radicals in the troposphere is an extremely demanding task due to their high reactivity, short lifetime and tiny concentrations. The first two decades after the discovery of atmospheric free radicals such as OH, HO_2_, RO_2_ and NO_3_, etc. were spent on instrument development worldwide. Only since the middle of the 1990s were the first generation of field-deployable instruments like DOAS, LIF and CIMS made available for the measurement of OH in a few groups in the USA and Europe. In China, the pioneer scientists had already well recognized the importance of atmospheric radicals in the early 1980s during the study of photochemical smog in Lanzhou. Also, after two decades of instrument development, the first successful measurement of OH and HO_2_ radicals was realized in rural Guangzhou and Beijing in summer 2006 in the framework of PRIDE-PRD through collaboration with Forschungszentrum Juelich (Germany) shortly after the first Asia urban OH measurement in Tokyo 2004. In the data analysis of the HOx observations in rural Guangzhou and Beijing and a recent campaign in rural NCP, we uncovered that the current tropospheric chemical mechanisms cannot explain the OH radical concentrations in China, which strongly underestimated the OH concentrations and the local ozone production for the low and high NO_x_ range, respectively. The new knowledge would mean that the use of current chemical mechanisms—Carbon Bond Mechanism (CBM) and SAPRC—in air-quality models is subject to large uncertainties for the diagnosis or prediction of air-pollution processes.

Since the establishment of the PKU-LIF instrument in 2014, new field studies on the investigation of free-radical chemistry have been extensively conducted in NCP and PRD again with the routine application of the chemical modulation method to ensure the OH measurement quality as suggested [89]. In the context of the global study on the radical chemistry, the recent field studies in China tell us that:

- the study of the unrecognized OH-regeneration mechanism needs to be continued after the quantification of the OH measurement interference problem in the near future;
- the strong underestimation of the local ozone production rate for the high NO_x_ air masses requires to be addressed more urgently with the recently available detection method—the selective detection of HO_2_ and the detection of RO_2_ by LIF techniques and even the further development of the ROx detection by PERCA may be rethought again. This is of central importance, since ozone pollution is becoming more and more serious for the many urban areas in China.
- very active night-time chemistry is probed due to the presence of both significant HOx concentrations as well as that of high night-time concentrations of NO_3_ and N_2_O_5_; this active night-time chemistry could influence the simulation of O_3_ and fine particles on the regional scale through many schemes like the removal of NO_x_, production of organic nitrates and activation of Cl chemistry, etc.
- due to fast oxidation of gaseous pollutants, NPF events in China have taken place under high CS conditions compared to the clean atmosphere globally. The efficient nucleation in polluted atmosphere greatly contributes to the number concentration of CCN, as well as haze formation. Comprehensive field measurement showed that the haze formation typically includes two distinct secondary aerosol formation process, namely efficient nucleation, and fast and continuous growth.

Overall, radical chemistry is key for the removal of primary pollutants and the production of secondary air pollution (e.g. PM_2.5_ and O_3_); effective control of Chinese air pollution relies on advancing knowledge on the atmospheric radical chemistry in China.
